# Body Mass Index and *Helicobacter pylori* among Obese and Non-Obese Patients in Najran, Saudi Arabia: A Case-Control Study

**DOI:** 10.3390/ijerph15112586

**Published:** 2018-11-19

**Authors:** Ali M. Al-Zubaidi, Abdo H. Alzobydi, Saeed A. Alsareii, AbdulazizTurky Al-Shahrani, Naweed Alzaman, Saba Kassim

**Affiliations:** 1 Department of Medicine, Endoscopy Unit, King Khalid Hospital, Najran 1120, Saudi Arabia; 2 Department of Surgery, Bariatric Surgery, King Khalid Hospital-Najran 1120, Saudi Arabia; alzobydi@yahoo.com; 3 Department of Surgery, Najran University Medical College and Hospital, Najran 1988, Saudi Arabia; alsareii997@hotmail.com; 4 Department of Internal Medicine; Najran University Medical College and Hospital, Nagran 1988, Saudi Arabia; dr.abdulaziz2012@hotmail.com; 5 Department of Internal Medicine, Taibah University Medical College, Al-Madinah Al-Munawwarah 42353, Saudi Arabia; naweedalzamanmd@gmail.com; 6 Department of Preventive Dental Sciences, Taibah University Dental College and Hospital, Al-Madinah Al-Munawwarah 42353, Saudi Arabia; saba262003@gmail.com

**Keywords:** obesity, endoscopy, *Helicobacter pylori*, BMI, Saudi patients

## Abstract

*Objective*: We examine obese and non-obese patients with respect to Helicobacter pylori (*H. pylori*) positive-infection (HPPI) and associated factors, specifically body mass index (BMI). *Methods*: This study took place in the Department of Endoscopy of a central hospital in the Najran region of Saudi Arabia (SA). A total of 340 obese Saudi patients (BMI ≥ 30 kg/m^2^) who had undergone diagnostic upper endoscopy before sleeve gastrectomy, were compared with 340 age and gender-matched control patients (BMI < 30 kg/m^2^) who had undergone diagnostic upper endoscopy for other reasons. Data collected included diagnosis of HPPI. Descriptive and multivariable binary logistic regression was conducted. *Results*: Mean patient age was 31.22 ± 8.10 years, and 65% were males. The total prevalence of HPPI was 58% (95% CI = 54–61%) with obese patients presenting significantly more HPPI than non-obese patients (66% vs. 50%, OR = 1.98, 95% CI = 1.45–2.70, *p* < 0.0005). Age and gender did not associate significantly with HPPI (*p* = 0.659, 0.200, respectively) and increases in BMI associated significantly with increases in HPPI (*p* < 0.0005). BMI remained a significant factor in HPPI when modelled with both age and gender (OR = 1.022, 95% CI = 1.01–1.03, *p* < 0.0005). *Conclusions*: Within the limitations of this study, the significance of HPPI in obese Saudi patients residing in the Najran region in SA was demonstrated alongside the significance role of BMI in HPPI.

## 1. Introduction

Helicobacter pylori (*H. pylori*) is one of the most common infections affecting the epithelial lining of the stomach [1]. It is correlated with antral gastritis, peptic ulcers and promotes gastric malignancies [2]. There were approximately 4.4 billion individuals with *H. pylori* infection worldwide in 2015 [1]. *H. pylori* infection is prevalent in developing countries [1] and risk factors for *H. pylori* including age and socioeconomic status [2].

There is ongoing debate over the relationship between obesity and *H. pylori* infection, within the acknowledgment that the etiology of obesity is far more complex [3,4,5]. A recent ecological review of several cross sectional studies found an inverse correlation between *H. pylori* prevalence and rate of overweight/obesity in countries of the developed world i.e., increase in *H. pylori* positive infection associated with reduction in obesity [6]. This was further corroborated with Intervention studies reporting that eradication of *H. pylori* was associated with significant weight gain as compared to subjects with untreated *H. pylori* [7,8]. In contrast, observational or clinical studies from developing countries reported a linear relationship between *H. pylori* positive infection and obesity [9,10,11].

Saudi Arabia has the highest obesity and overweight prevalence rates in the Middle East which is linked to multiple factors including adapting a westernized life style [12]. The prevalence of *H. pylori* within the Saudi Arabian population and related factors remains unknown and most available data is reported from medical care settings with a range of prevalence between 33–85% [9,13,14,15,16,17]. A study randomly selecting university students reported a prevalence of 35% for *H. pylori* [18]. Specifically, high *H. pylori* prevalence was reported among morbid obese patients who underwent upper endoscopy prior to bariatric surgery was 88% [9]. Yet the matching of this latter group with normal body weight patients to substantiate such findings within the context of Saudi Arabia has not been attempted specifically in the light of controversy of the association of *H. pylori* with obesity [6,7,8].

Morbid obesity has been widely treated with various type of bariatric surgery. Bariatric surgery includes laparo-scopic adjustable gastric bands (LAGB), lap band, the Roux-Y gastric bypass (RYGB) and sleeve gastrectomy or gastric sleeve [19]. Routine upper endoscopy with *H. pylori* screening and biopsies to rule out pathological abnormalities (e.g., gastritis, duodenitis, esophagitis, ulceration, hiatus hernia, …, etc.) is able to detect abnormalities in up to 91% of bariatric candidates [20] with a higher rate in patients with concomitant *H. pylori* infection [21,22,23,24].

The American Association of Clinical Endocrinologists/The Obesity Society/American Society for Metabolic and Bariatric Surgery guidelines [25] have not provided any clear indication about preoperative *H. pylori* screening and management but recommended *H. pylori* screening in patients belonging to high-prevalence areas and upper endoscopy in selected cases. On the other hand, the European guidelines [26] have recommended upper gastrointestinal endoscopy before bariatric surgery in both symptomatic and asymptomatic patients and to treat *H. pylori* infection and other abnormalities, which may cause postoperative complications.

In light of the limited literature to assess the prevalence of HPPI in patients undergoing bariatric surgery from the Middle East and North Africa (MENA) and specifically Saudi Arabia therefore, we aimed in this hospital-based study to determine the estimate of *H. pylori* positive infection (HPPI) in group of morbidly obese subjects undergoing sleeve gastrectomy and compare it to a match control group (age and gender) who had an upper endoscopy in the same period and same setting with different indications but had normal body weight. Based on the conflicting results of the current literature, we hypothesized, after controlling for age and gender, that we will be able to find out whether there is correlation between HPPI and obesity.

## 2. Material and Methods

### 2.1. Study Design and Setting

A case-control study design was adopted in the department of endoscopy at a central hospital (King Khalid) which received all medical referrals in Najran region southwest of Saudi Arabia.

### 2.2. Subjects’ Recruitment and Inclusion and Exclusion Criteria

A consecutive test group consisted of 340 obese patients; defined with a BMI of ≥30 kg/m^2^ with a serious comorbidity (e.g., diabetes mellitus, ischemic heart disease, obstructive sleep apnea) or with a BMI of ≥40 [27] who had undergone diagnostic upper endoscopy before sleeve gastrectomy were enrolled into the study over the period between January 2013–December 2014. Over the same period, a similar number of 340 patients; with BMI of <30 kg/m^2^ who had undergone diagnostic upper endoscopy in the same hospital and department due to other reasons and complain (e.g., epigastric pain and dyspepsia) were selected as controls with approximately 1:1 ratio test to control. Both groups self-reported neither tested nor treated for *H. Pylori* in the past 5 years.

We excluded patients with advanced liver disease, malignancy, renal failure, those taking bismuth subcitrate, history of taking eradication therapy of HPPI within six months, with acute infection and, gastro intestinal bleeding or patients with history of proton pump inhibitor (PPI) or antibiotics intake 2 weeks before the endoscopic procedure as these are known to may lead to false negative results for HPPI test.

### 2.3. Variables and Clinical Procedures

The data for both groups included the demographics (age, gender), medication use, laboratory results (e.g., CBC, coagulation profile, RFT, LFT, Cortisol, TFT, blood sugar, lipid profile), endoscopic and histologic finding (e.g., Gastritis, PUD, GERD, Hiatus Hernia). All patients underwent the endoscopic procedure with standard technique (using Olympus GF260, 9.6 mm diameter scope) to assess for HPPI test. Patient were lying in the left lateral position with a mouth piece placed and their throat sprayed with adequate xylocaine. Two antral biopsies were taken from antrum and lower body for each patient, as per the routine practice of the endoscopy unit, and submitted for histologic evaluation. No complication related to endoscopy was reported in any subject. The specimens were stained by hematoxylin and eosin stain or methylene blue. The laboratory investigator was blinded to the sample status to avoid bias.

Body mass index (BMI) is a simple index of weight-for-height that is commonly used to classify overweight and obesity in adults. It is defined as a person’s weight in kilograms divided by the square of his height in meters (kg/m^2^). BMI greater than or equal to 25 is considered to be overweight, and BMI greater than or equal to 30 is considered to be obesity [28]. As for body mass index a digital scale was used to measure the weight of patient to the nearest 0.1 kg and height was considered in a standing position without shoes and in light clothing.

### 2.4. Ethical Considerations

The study’s proposal was reviewed and guaranteed clearance from the Ethical Committee of King Khalid Hospital, Najran (H-11-N081, Ali Mothanna Al-Zubaidi, 2012). The study was conducted in accordance with the Declaration of Helsinki wherein respondents were briefed about the study and the anticipated benefits of participating in the study. An information sheet explaining the aims and objectives of the study was made available for each respondent. Voluntary participation was emphasized and withdrawal from the study at any time without giving reason(s) would not disadvantage the respondent from receiving any treatment. An informed consent form was obtained before participating in the study and confidentiality of the information obtained was assured.

### 2.5. Statistical Analysis

Descriptive analysis was conducted and continuous variables were reported using Mean ± SD and for categorical variables we used frequencies with percentages. Unpaired *T*-Test and Chi squared tests were performed to explore the relationship of continuous and categorical variables with the dependent variable (‘HPPI’ (*H. pylori* positive infection) and ‘HPNI’ (*H. pylori* negative infection)). Binary logistic regression analysis was used to model the relationship of BMI after controlling for age and gender with the dependent variable. A *p* value of <0.05 was considered significant. The statistical analyses aforementioned were performed withe SPSS Software version 16.0 (SPSS Inc., Chicago, IL, USA).

## 3. Results

### 3.1. Sample Characteristics and Distribution of HPPI between and within Obese and Non-Obese

The demographic characteristics for both groups are shown in Table 1. There were no significant differences in the age of both obese and none obese patients (*p* = 0.307) with a male predominance in both groups (66.5% and 64.4%). The total mean of BMI for both groups was 35.57 ± 13.97 and there were statistical significant differences in the mean BMI between the obese and none obese (BMI = 23.13 ± 3.80 for none obese vs. 48.04 ± 8.10 for obese, *p* < 0.0005). As demonstrated in Table 1, the total prevalence of *H. pylori* positive-infection (HPPI) was 58% (95% CI = 54–61%). A statistical significant difference in HPPI between obese and non-obese was observed (225 (66.2%) for obese vs. 169 (49.7%) non-obese, OR (95% CI) = 1.98 (1.45–2.70), *p* < 0.0005). Within the gender groups, females were found to slightly predominate in HPPI. Males and females with respect to HPPI among obese patients was non-significant (146 (64.6%) vs. 79 (69.3%), OR = 1.23 (0.76–2.00), *p* = 0.388) and likewise among non-obese (104 (47.5%) vs. 65 (53.7%), OR (95% CI) = 1.28 (0.82–2.00), *p* = 0.271). However, between groups, Table 1 shows that both males and females were statistical significantly different in HPPI (*p* < 0.0005, 0.014, respectively). Unpaired *T*-Test and Chi squared tests revealed that age and gender did not associate with HPPI (*p* = 0.659, 0.200, respectively) and increases in BMI associated significantly with increases in HPPI (*p* < 0.0005).

### 3.2. Multivariable Analyses of Adjusted Factors of HPPI

Multivariable binary logistic regression revealed that BMI remained significant factor in HPPI when modelled with both age and gender (OR = 1.022, 95% CI = 1.01–1.03, *p* < 0.0005) (Table 2).

## 4. Discussion

To the best of our knowledge, this case-control study is the first conducted in this region of Najran, SA that considered the none-modifiable factors (age and gender) in modelling relationship between obesity and *H. pylori* positive infection. The key finding was that increased prevalence of HPPI was associated with increased BMI levels, both with and without adjustment for none modifiable factors (age and gender). The findings of this study added to the current debate about the relationship of obesity with HPPI. A recent review showed a significantly inverse correlation between *H. pylori* prevalence and rate of overweight/obesity in countries of the developed world. Thus, the obesity endemic observed in the Western world was attributed to the gradual decrease of the HPPI [6]. These later results were also supported with studies in certain areas in Asian region e.g., Taiwan, [29].

However, our study findings were in consensus with previous studies reported within SA [9] and elsewhere [11,30]. In Turkey a prevalence of 57.2% of HPPI in Turkish obese subjects compared to 27.0% in normal body weight was found and this further supported our finding [31].

The estimate of HPPI among obese patients who underwent sleeve gastrectomy in this study sample was 66% which was in mid-range (50–88) of previous studies conducted in in SA [13,32,33] and higher than a study among young medical students that showed a low prevalence of 35% [18]. Different study design, participants’ recruitments and clinical investigation may reflect such variations in the estimate of HPPI among obese subjects. In addition, the role of the difference in geographic, economic or environmental factors should be considered. Notably, our study was comparable to one US study which found a higher prevalence rate of 61% in morbidly obese patients compared to 48% in the control group [34] though this later used *H. pylori* serologies, while our study used histopathology diagnosis. With respect to the role of age and gender in HPPI, they were found to be non-significantly associated with HPPI and these findings were in consensus with other studies reported in the region [15,33] and elsewhere [35].

As for the strengths of this study, firstly our study findings have substantiated the relationship of obesity with *H. pylori* among obese patients who were matched with none obese patients consecutively recruited in the same setting with a standardized investigation procedures i.e., internal validity plausible. Secondly, this was a hospital-based case-control as such more prone to bias, specifically when selecting participants. However, we were in line with what has been reported previously [30] patients underwent a gastroscopy and received a histological examination of gastric mucosal biopsies as a standard diagnostic examination before bariatric surgery. Thus, the majority of the examined patients were asymptomatic which excludes a strong selection bias toward HPPI. Our study results were of validity as the inclusion and exclusion criteria was adequate to rule out any confounding effects; e.g., use of antibiotics [34]. The enrolment of patients from the same region and in one Centre (Najran Hospital) is another strength of our study, the difference in the estimate of *H. pylori* between the two groups was less likely to represent a differences in geographic prevalence of *H. pylori* as reported elsewhere [34]. Finally, the feasibility of the study with respect of time and resources was additional strength. As for the limitations, the sampling method adopted precludes the generalizability of the study findings to individuals outside of this setting. The patients self-reported of neither tested nor treated for *H. pylori* was not objectively validated, therefore, recall bias was possible. The impact of the latter on the validity of the results might be inevitable. Importantly, in SA the role of race in HPPI should be investigated as elsewhere [29]. The use of the concept of nationality is not sufficient as many Saudi national of different ethnicity/origin backgrounds that presumably influence HPPI. The contribution of other environmental and host factors [36] which has not been controlled for in this study should be considered in future research.

## 5. Conclusions

Within the limitations of this study, the significance of HPPI in obese Saudi patients residing in the Najran region in SA was demonstrated alongside the significance role of BMI in HPPI. Further research should consider a comprehensive and context specific factors (e.g., environmental and host) contribution to clarify the association of obesity with HPPI.

## Figures and Tables

**Table 1:** Characteristics of obese (*N* = 340) and none-obese (*N* = 340) and distributions of HPPI between groups and gender.

Variables	Mean ± SD or *N* (%)		OR (95% CI)	*p*-Value
	Obese (Cases)	Non-Obese (Controlled)		
**Age**	31.54 ± 8.27	30.90 ± 7.93	-	0.307
**Gender**				
Male	226 (66.5)	219 (64.4)		
Female	114 (33.5)	121 (35.6)	0.93 (0.67–1.25)	0.572
**BMI**	48.04 ± 8.10	23.13 ± 3.80	-	0.0005
**HPPI**	225 (66.2)	169 (49.7)	1.98 (1.45–2.70)	0.0005
**HPPI in male**	146 (58.4)	104 (41.6)	2.02 (1.38–2.95)	0.0005
**HPPI in female**	79 (54.9)	65 (45.1)	1.95 (1.14–3.32)	0.014

SD = Standard deviation; F (%) = Frequency with percentage; HPPI = *H. pylori* positive infection; OR (95% CI) = Odd ratio with 95% confidence interval.

**Table 2:** Adjusted model for the association of BMI with HPPI.

Explanatory Variables	Wald	AOR (95% CI) *	*p*-Value
**Age**	0.371	0.99 (0.98–1.03)	0.542
**Gender**			
Male		1	
Female	2.11	1.28 (0.92–1.77)	0.146
**BMI**	13.67	1.02 (1.01–1.03)	0.0005

* AOR (95% CI) = adjusted odd ratio and 95% confidence interval.
